# Analysis of *MsTERT* Gene Expression Profile in Alfalfa (*Medicago sativa*) Indicates Their Response to Abiotic Stress and Seed Aging

**DOI:** 10.3390/plants12102036

**Published:** 2023-05-19

**Authors:** Shoujiang Sun, Wen Ma, Peisheng Mao

**Affiliations:** Forage Seed Laboratory, College of Grassland Science and Technology, China Agricultural University, Beijing 100193, China; shoujiangsun@cau.edu.cn (S.S.); mmw21@cau.edu.cn (W.M.)

**Keywords:** alfalfa, telomerase reverse transcriptase, seed aging, abiotic stress, *MsTERT* gene

## Abstract

Seed aging is always taken as a crucial factor for vigor loss due to delayed seed germination and seedling growth, which limits hay production. Many studies have found that telomeres are closely related to abiotic stress and seed vigor. However, the molecular mechanism of telomeres’ response to abiotic stress, seed vigor, and the maintenance mechanism of plant telomere homeostasis still remain unclear. Alfalfa (*Medicago sativa*) enjoys the title of “King of Forage”, and is an important protein forage for the dairy industry as planted in the world. This comprehensive investigation was performed to explore the molecular characterization, phylogenetic relationship, and gene expression analysis of *MsTERT* under abiotic stress and during seed aging in alfalfa. In this study, *MsTERT* was identified from the ‘Zhongmu 1’ alfalfa genome and encoded a coding sequence (CDS) of 3615 bp in length, consisting of telomerase- RNA-Binding Domain (RBD) and Reverse Transcriptase (RT) domains, 1024 amino acids, an isoelectric point of 9.58, and a relative molecular mass of 138.94 kD. Subcellular localization showed that *MsTERT* was mainly localized in the nucleus and mitochondria. The results of the expression profile showed that *MsTERT* was observed to respond to various stress conditions such as salt (100 mmol/L NaCl) and drought (20% PEG 6000). Furthermore, exogenous hormones IAA, ABA, and GA_3_ showed the potential to affect *MsTERT* expression. Additionally, *MsTERT* also responded to seed aging. Our results revealed a marginal but significant association between relative telomere length, *MsTERT* expression, and seed germination percentage, suggesting that the length of telomeres was shortened, and expression of *MsTERT* decreased with alfalfa seed aged. These results provide some evidence for the hypothesis of relative telomere length and/or TERT expression serving as biomarkers of seed aging. Although this finding is helpful to offer a new way to elucidate the molecular mechanism of vigor loss in alfalfa seed, further investigation is required to elucidate the molecular mechanism by which the *MsTERT gene* regulates seed vigor.

## 1. Introduction

Alfalfa (*Medicago sativa*), with the reputation of “King of herbage”, is a perennial leguminous plant that has mostly grown in the 14 northern provinces of China for more than two thousand years [1]. Seed aging, saline-alkali, and drought are the vital factors that affect alfalfa seed germination, seedling establishment, and plant growth, which limit its productivity and survival in the whole area. Many studies have found that telomeres are closely related to seed aging and responses to abiotic stress. Telomeres are a special structure located at both ends of chromosomes, which are the “clock” that controls the number of cell divisions [2]. Eukaryotic telomeres are composed of specific repetitive DNA sequences and binding proteins [3]. Since their deletion leads to the fusion of chromosome ends and loss of genes, it is thought to be critical for maintaining genome stability. In plants, telomeres are essential for normal development and differentiation [4]. Although some discoveries have been made in research on plant telomeres in recent years, the exact mechanisms of the telomeres’ responses to abiotic stress and seed aging, as well as the maintenance mechanism of plant telomere homeostasis, still remain unclear.

Telomerase has telomerase RNA (TER) and TERT two core subunits [5], both associated with telomerase-associated proteins (TAP), together make up the telomerase structure. TERT is the key gene that regulates telomerase activity, and reverse transcriptase is its speed-limiting subunit [6]. TERT also has other non-telomeric functions, such as reducing ROS production; increasing the rate of breathing in cells; protecting mtDNA by directly combining with mitochondrial tRNA interactions [7]; and increasing mitochondrial membrane potential (mtDNA) [8]. Oguchi et al. [9] cloned and identified the homology gene *AtTERT* in *Arabidopsis thaliana* on the basis of human *hTERT* gene prediction. In 2004, Oguchi et al. [10] discovered the alternative splicing transcripts of five *OsTERT* genes in rice. Sun et al. [11] analyzed the structure and function of *AmTERT* in *Ammopiptanthus Mongolicus* and the expression of *AmRERT* in root, stem, and leaf tissue under salt, drought, heat, and low-temperature stress. Now, the non-telomeric functions, especially the function of TERT to reduce the generation of ROS and improve the stress tolerance of plants, have been paid more and more attention. Correia et al. [12] found that the telomerase activity and the expression of AtTERT increased at first and then decreased under salt stress and H_2_O_2_ stress in Arabidopsis; TERT in sunflower leaves was down-regulated by salt stress [13]; gene silencing of GlTERT in *Ganoderma lucidum* showed that the growth rate decreased, the ROS level increased, and the activity of antioxidant enzymes decreased [1]; the expression of *AmTERT* in the roots and leaves of *Ammopiptanthus mongolicus* seedlings increased under salt, drought, heat, and low-temperature stress.

This present study identified the TERT in the alfalfa genome of “Zhongmu No. 1”, and analyzed its expression profiling under drought stress, salt stress, hormone treatment, different tissues, and different vigor seeds by qPCR. We hypothesized that relative telomere length and/or TERT expression can serve as biomarkers of seed aging. This work provides valuable information on potential biomarkers of alfalfa seed aging and contributes to our understanding of this process. Additionally, these findings can be used for genetic improvement of stress resistance and seed vigor in alfalfa.

## 2. Results

### 2.1. Identification and Chromosomal Mapping of MsTERT Gene

**Figure 1:**
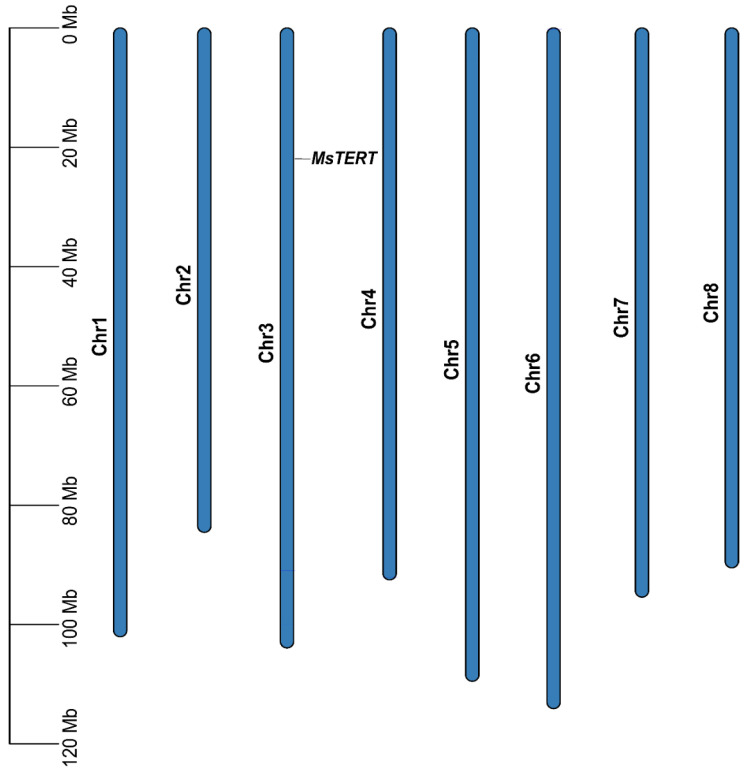
Chromosomal mapping of *MsTERT* genes on the eight alfalfa chromosomes. The chromosome number is denoted on the middle of each chromosome. The scale is marked in megabases (Mb).

In order to obtain the *MsTERT* protein of alfalfa, we used the *Medicago truncatula* MtTERT protein as a query in a BLASTP search against the *M. sativa* genome of “Zhongmu No. 1”, which was downloaded from the FIGSHARE database (https://figshare.com/, accessed on 11 September 2020). One *MsTERT* gene was identified in *M. sativa* and distributed in the alfalfa chromosome 3 (Figure 1). Additionally, we looked into the physico-chemical properties of *MsTERT* proteins, including the amino acid composition, molecular weight, and theoretical pI. The CDS sequence of *MsTERT* was 3615 bp, which encoded 1024 amino acids, the molecular weight was 138.94 kDa, and the isoelectric point is 9.

### 2.2. Physico-Chemical Properties of MsTERT Protein

In order to understand the physico-chemical properties of *MsTERT* proteins, we computed parameters that include the prediction of a signal peptide, protein transmembrane structure, hydrophobicity, and phosphorylation. The prediction of the protein signal peptide sequence shows that there is no signal peptide sequence in *MsTERT* amino acid, and the prediction of the protein transmembrane structure shows that there is no transmembrane structure, which means that *MsTERT* protein is not a transmembrane protein. The hydrophobicity of amino acids was predicted, which indicated that *MsTERT* was a hydrophilic protein.

Phosphorylation is a common regulatory mode of protein function and activity. In total, 122 potential phosphorylation sites of *MsTERT* protein were found, including 85 tryptophan (Ser) phosphorylation sites distributed across almost the whole polypeptide chain, twenty-eight threonine (Thr) phosphorylation sites, and nine tyrosine (Tyr) phosphorylation sites.

### 2.3. Phylogenetic and Motif Pattern Analysis of TERT Proteins

**Figure 2:**
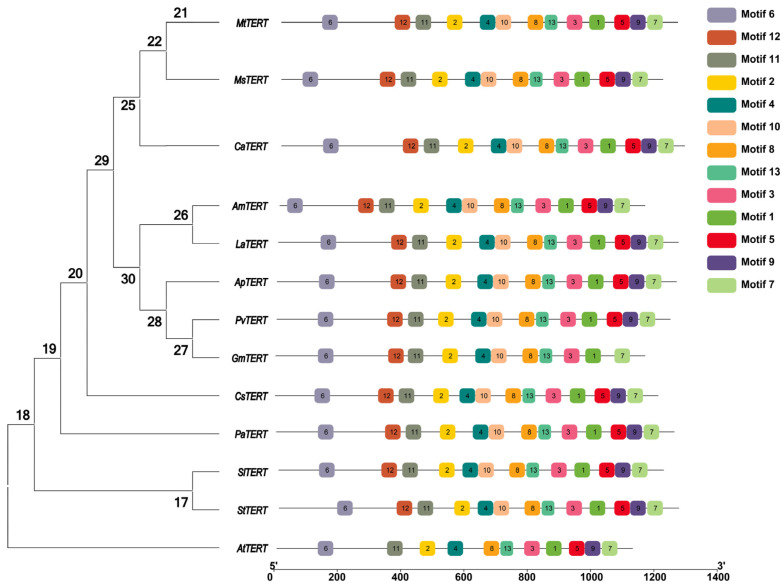
Analysis of TERT conserved domains in different species.

In this study, 13 species, including *Medicago truncatula*, *Medicago sativa*, *Cicer arietinum*, *Ammopiptanthus mongolicus*, *Lupinus micranthus*, *Abrus precatorius*, *Phaseolus vulgaris*, *Glycine max*, *Cucumis sativus*, *Armeniaca mume*, *Solanum lycopersicum*, *Solanum tuberosum,* and *Arabidopsis thaliana* were selected to construct an evolutionary tree through MEGA 8.0 software *using* N J method (bootstrap = 1000) (Figure 2). The results showed that the *MsTERT* has high homology with *Medicago truncatula*, *Medicago sativa,* and *Cicer arietinum* but is far away from *Armeniaca mume*, *Solanum lycopersicum*, *Solanum tuberosum,* and *Arabidopsis thaliana*. The online MEME search tool (http://meme-suite.org/, accessed on 11 December 2022) was used to further look into the conversation of TERT proteins. TBtools software (https://github.com/CJ-Chen/TBtools/v1.120, accessed on 16 May 2023) was used to edit the motif pattern and gene structure diagrams [14]. In addition, 13 species, including *Medicago truncatula*, *Medicago sativa*, *Cicer arietinum*, *Ammopiptanthus mongolicus*, *Lupinus micranthus*, *Abrus precatorius*, *Phaseolus vulgaris*, *Glycine max*, *Cucumis sativus*, *Armeniaca mume*, *Solanum lycopersicum*, *Solanum tuberosum,* and *Arabidopsis thaliana* were selected to analyze the conservation of TERT protein. In total, 13 conserved structural domains were identified (Figure 2), with all 12 TERT proteins sharing the same structural domain, except for AsTERT, which does not have conserved structural domain number 12.

### 2.4. Structural Analysis of MsTERT

**Figure 3:**
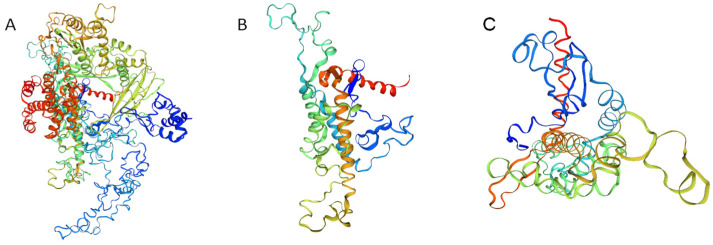
Three-dimensional structure diagram model of *MsTERT*. Note: (**A**): The three-dimensional structural model of *MsTERT*; (**B**): The three-dimensional structural model of the telomerase RNA binding domain (TRBD) of *MsTERT*; (**C**): The three-dimensional structural model of the reverse transcriptional activation region (RT) of *MsTERT*.

By using the SWISS-MODEL to predict the three-order conformation of *MsTERT* (Figure 3), it is found that *MsTERT* has reverse transcriptase activity domain (RT) and Telomerase RNA binding domain (Telomerase), as well as AtTERT, MtTERT, CaTERT, etc. It is concluded that the *MsTERT* protein has a typical TERT structure.

### 2.5. MsTERT–GFP Protein Was Localized in the Nucleus and Mitochondrion

**Figure 4:**
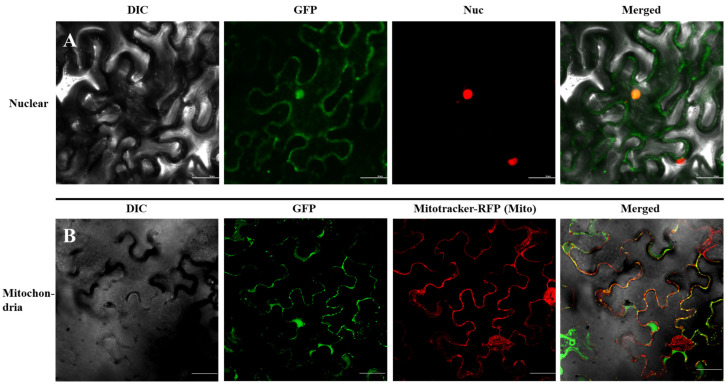
Subcellular localization of MsTERT–GFP in *Nicotiana tabacum*. (**A**) At-hook-RFP—which was used to indicate the location of nucleus—and MsTERT–GFP were injected into tobacco leaves. (**B**) Tobacco leaves transfected with MsTERT–GFP were stained with Mitotracker which was used to indicate the location of mitochondria. After 24 h of expression, the leaves were observed by confocal laser scanning microscope. Scale bar 50 µm.

We re-evaluated the possible targeting signals using the PSORT program (version WoLF, http://psort.hgc.jp/, accessed on 11 December 2022), and signals for the nucleus, mitochondrion, and cytoplasm were predicted. To investigate the intracellular localization of *MsTERT* in plant cells, GFP was fused to the C-terminus of *MsTERT* to construct an expression vector for transient expression in tobacco leaves. We co-expressed MsTERT–GFP in tobacco leaves with a fluorescent marker protein that would mark the nucleus [At-hook-RFP red fluorescent protein]; the fluorescence of MsTERT–GFP fusion protein colocalized with the nucleus marker At-hook-RFP (Figure 4A), which demonstrated a clear localization of *MsTERT* in the nucleus. To further document the localization of *MsTERT*, we co-expressed the MsTERT–GFP with fluorescent marker proteins for mitochondria and stained the mitochondria with Mitotracker. Our results showed a fluorescent signal in the mitochondria (Figure 4B), which demonstrated a clear localization of *MsTERT* in the mitochondrion. Surprisingly, our test results are consistent with the predicted results.

### 2.6. Identification of Cis-Elements in the Promoter Region of MsTERT Genes

The promoter sequence of 2000 bp upstream of the *MsTERT gene* was analyzed by using the Plant CARE online website (Table 1). The results showed that there were the promoter and enhancer regions commonly found in the promoter; promoter elements in the upstream 30 bp transcriptional initiation region; the promoter region itself had a promoter and enhancer region; there was a promoter region in the upstream 30 bp transcriptional initiation region; and it also has a drought-induced MYB binding site, a gibberellin-responsive cis-regulatory element, and plant growth hormone-responsive elements. This suggests that the *MsTERT* gene has non-telomere function and may be involved in abiotic stress regulation.

### 2.7. Expression Analysis of MsTERT Genes under Hormone Treatment

**Figure 5:**
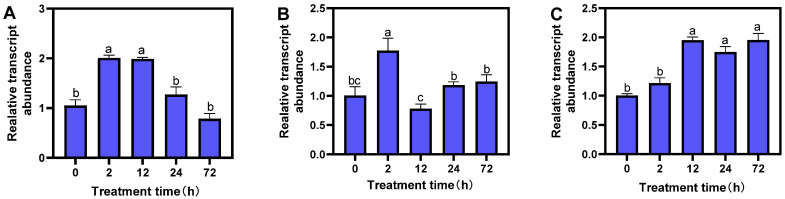
Expression profiling of *MsTERT* gene in response to three hormones of IAA (**A**), ABA (**B**), and GA (**C**). Expression levels are normalized to Ms-ACTIN, and the letters represent statistical significance among the treatments and the vertical bars represent the ±SEM at *p* < 0.05 level for three replicates. The mean values sharing same letters, obtained from Duncan test, are not different significantly.

From the 2000 bp promoter sequence of the *MsTERT* gene upstream analyzed by Plant CARE online, we found that there are many kinds of cis-regulatory elements and plant growth hormone response elements in the promoter region. In order to explore the response pattern of the *MsTERT* gene to different hormones (IAA, ABA, and GA_3_), three-leaf alfalfa seedlings were treated with different hormones for 0, 2, 12, 24, and 72 h, respectively; quantitative Primer MsTERT-F/MsTERT-R (Table 2) was used to analyze *MsTERT* gene expression. The results showed that the expression level of the *MsTERT* gene increased at first and then decreased with the extension of treatment time under 1 mmol·L^−1^ IAA treatment, and reached the maximum after 12 h of treatment, when the difference was significant (*p* < 0.05), which then decreased (Figure 5A). After treatment with 1 mmol·L^−1^ ABA, the *MsTERT* gene expression increased at first and then decreased with the extension of treatment time, and reached the maximum after 2 h of treatment, the difference was significant (*p* < 0.05) compared with 0 h, which then decreased gradually (Figure 5B). After 1 mmol·L^−1^ GA_3_ treatment, the expression level increased gradually with the prolonging of treatment time, and the expression level reached the maximum after 12 h treatment, the difference was significant compared with 0 h treatment (*p* < 0.05), the amount of expression remained stable (Figure 5C).

### 2.8. Response of MsTERT to Drought and Salt Stress

**Figure 6:**
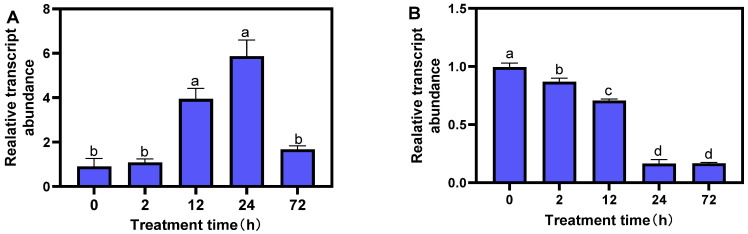
Expression profiling of MsTERT gene in response to drought (**A**) and salt (**B**) stress treatments. Expression levels are normalized to Ms-ACTIN, and the letters represent statistical significance among the treatments and the vertical bars represent the ±SEM at *p* < 0.05 level for three replicates. The mean values sharing same letters, obtained from Duncan test, are not different significantly.

In order to explore the response pattern of *MsTERT* gene to different abiotic stresses, alfalfa seedlings at the three-leaf stage were treated with drought (20% PEG 6000) and salt (100 mmol·L^−1^ NaCl) for 0, 2, 12, 24, and 72 h, respectively; quantitative Primer MsTERT-F/MsTERT-R was used (Table 2). The expression of the *MsTERT* gene was analyzed. The results showed that the *MsTERT* gene expression increased at first and then decreased with the prolonging of treatment time under 20% PEG treatment, and the *MsTERT* gene expression remained stable for 2 h. After 12 h treatment, the expression level gradually increased and reached the maximum after 24 h treatment, the difference was significant (*p* < 0.05), and then the expression level decreased (Figure 6A). After 100 mmol·L^−1^ NaCl treatment, the expression of the *MsTERT* gene decreased gradually with the extension in treatment time, and the expression level of the *MsTERT* gene decreased sharply after 24 h treatment (*p* < 0.05), then the amount of expression remained stable (Figure 6B).

### 2.9. Tissue-Specific Expression Analysis of MsTERT Gene

**Figure 7:**
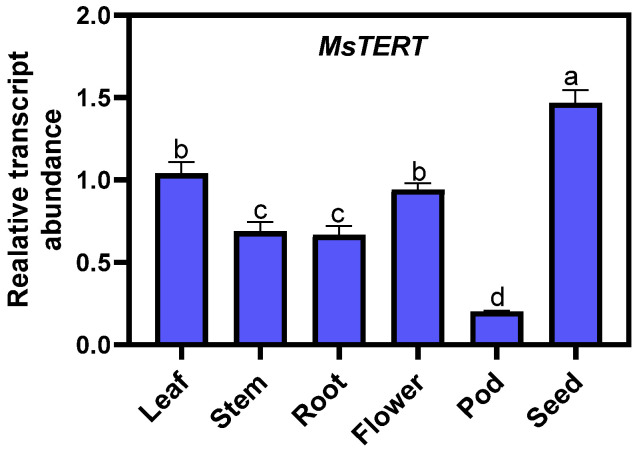
Expression profiling of *MsTERT* gene in different tissues of *M. sativa.* The relative expression was calculated using transcription level of leaf as reference, and the letters represent statistical significance among the treatments and the vertical bars represent the ±SEM at *p* < 0.05 level for three replicates. The mean values sharing same letters, obtained from Duncan test, are not different significantly.

The qPCR was used to analyze the expression of *MsTERT* in roots, stems, leaves, and flowers at full flowering stage, pods at yellow ripening stage, and seeds harvested at full ripening stage. The detection results showed that (Figure 7), the expression of *MsTERT* in different organs was seed > leaf > flower > stem > root > pod; the expression level in seeds was the highest, which was approx. two to four times that of other tissues, and was the lowest in the pods.

### 2.10. Response to Seed Aging Stress of MsTERT

**Figure 8:**
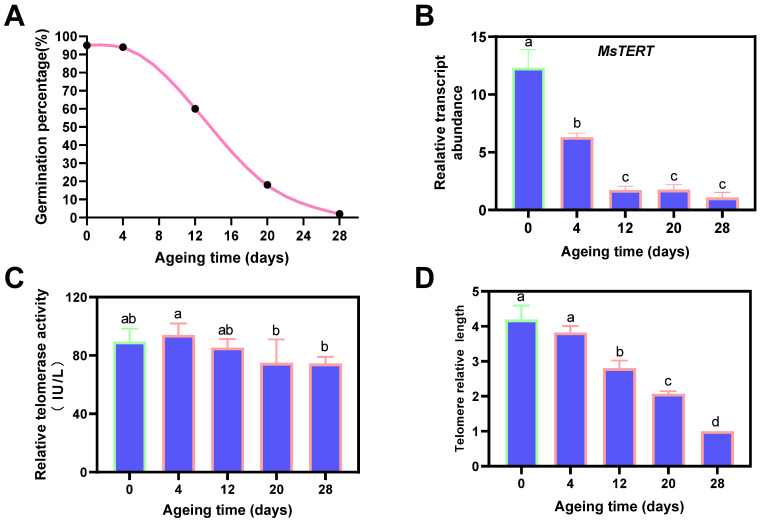
Influence of aging treatment on alfalfa seed germination (**A**), expression profiling of *MsTERT* gene (**B**), telomerase activity (**C**) and telomere length (**D**). The letters represent statistical significance among the treatments and the vertical bars represent the ±SEM at *p* < 0.05 level for three replicates. The mean values sharing same letters, obtained from Duncan test, are not different significantly.

The germination percentage of alfalfa seeds decreased gradually with the increase in aging days, decreased rapidly after aging for 4 days, and decreased to 0% after aging for 28 days (Figure 8A). The expression of *MsTERT* gradually decreased with the aging days, which decreased rapidly after aging for 4 days with a significant difference (*p* < 0.05) compared with 0 days. The differences between 4 days and 12, 20, and 28 days were significant (*p* < 0.05) (Figure 8B). The telomerase activity increased first and then decreased with the prolongation of aging time. At the beginning of aging, the telomerase activity increased and then gradually decreased. The telomerase activity reached the maximum value on the 4th day of aging. The differences between 4 days and 20 and 28 days were significant (*p* < 0.05) (Figure 8C). Telomere length was gradually shortened with aging days, and there were significant differences between 0 days and 12, 20, and 28 days (*p* < 0.05) (Figure 8D).

### 2.11. Correlation Analysis between the Five Indicators

**Figure 9:**
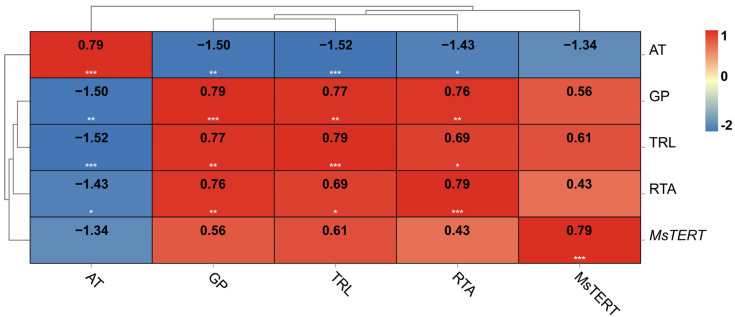
Correlation of seed vigor with *MsTERT* expression, relative telomere length, and relative telomerase activity. AT, GP, TRL and RTA represent aging time, germination percentage, telomere length and telomerase activity, respectively.The relative expression of *MsTERT* was plotted against seed germination at 0, 4, 12, 20, and 28 days of aging treatment. The numbers in the rectangle represent the Pearson correlation coefficient. *, ** and *** represent significant correlation at the *p* < 0.05, *p* < 0.01 and *p* < 0.001, respectively.

The results showed that the five indexes were highly correlated (Figure 9), and the germination percentage, as a representative index of seed vigor, was positively correlated with the relative telomerase activity, *MsTERT* expression, and telomere length; it was negatively correlated with aging days. However, the relative telomerase activity, *MsTERT* expression, telomere length, and germination percentage were negatively correlated with aging days.

## 3. Discussion

### 3.1. Identification of MsTERT Bioinformatics

The telomere DNA is composed of the repetitive DNA sequence of purine bases, which is extremely sensitive to reactive oxygen species (ROS), and needs to be repaired by telomerase after telomere DNA damage, therefore, the change in telomerase activity reflects the change in telomeres to some extent. Telomerase is a special kind of reverse transcriptase, which is composed of two core subunits of telomerase RNA and TERT Ribonucleoprotein, and has an important catalytic repair effect on a telomere’s loss, thus the telomere length maintains the integrity of the cell chromosome end structure and guarantees the cell normal metabolism. When telomerase activity is inhibited, telomeres shorten gradually during DNA replication during cell division, and shortening of telomere length to critical length leads to activation of the apoptosis pathway; thus, changes in telomere length and telomerase activity directly affect cell survival. TERT serves as the fundamental constituent of telomerase, and an investigation of its functionality facilitates an in-depth elucidation of the regulatory mechanism underlying telomerase activity. In this study, *MsTERT* was identified to have the same basic characteristics that are similar to other TERTs and includes the Telomerase RNA binding domain (telomere) and the TERT domain (RT). The promoter sequence contains components that respond to both hormonal and abiotic stresses. These structural characteristics are the basis of TERT’s responses to phytohormone regulation and abiotic stresses.

In summary, *MsTERT* has a TERT-conserved domain: telomerase RNA binding domain (TRBD) and telomerase reverse transcriptase activity domain (RT). At the same time, it also contains many potential kinase phosphorylation sites, which may be involved in the regulation of telomere and non-telomere functions of alfalfa telomerase. It also has non-telomere functions of stress protection and response to oxidative DNA damage as animal cells do. However, its specific stress response mechanism needs further study. The results of this study provide an important reference for further study on *MsTERT* in alfalfa growth and development, seed vigor control, and abiotic stress-resistance breeding.

### 3.2. MsTERT Response Patterns to Abiotic Stress and Hormone Treatments

Many studies have shown that TERT not only has telomeric DNA synthesis but also non-telomeric functions, such as inducing a DNA repair system, antagonizing apoptosis, and protecting mitochondrial integrity [15,16]. Haendeler et al. [17] studied human hTERT and found that ND1 and ND2 of mtDNA combined with hTERT inhibited the production of ROS by respiratory chain complex I, so as to maintain the activity of respiratory chain and avoid the damage of mitochondria induced by oxidative stress. In plants, TERT is also involved in abiotic stress regulation. The results of this study also showed that *MsTERT* also had non-telomeric functions. qPCR results showed that *MsTERT* was expressed in different degrees in alfalfa roots, stems, leaves, flowers, pods, and seeds (Figure 7). Yang et al. [18] found that the resistance of *E. coli* transformed with *AtTERT* under NaCl salt stress, osmotic stress, and hydrogen peroxide stress was enhanced. However, the resistance of *E. coli* transformed with AtTERT under low temperatures was reduced, indicating that *A. thaliana* AtTERT has the non-telomere function of resisting abiotic stress. It is considered that the telomerase of *A. thaliana* cells plays a certain role in resisting intracellular oxidative damage. In this study, alfalfa seedlings were treated with 20% PEG 6000 simulated drought and 100 mmol·L^−1^ NaCl salt stress, respectively. It was found that both treatments could induce the expression of *MsTERT*. The expression level was up-regulated in the initial stage of PEG treatment and decreased after 72 h, indicating that there may be a threshold for the ability of telomerase to resist drought stress. During 100 mmol·L^−1^ NaCl salt stress treatment, the expression of *MsTERT* was downregulated with the extension of treatment time, which may be a negative regulatory effect on salt stress (Figure 9). The maintenance of telomerase activity plays a very important role in protecting cells from stress and maintaining the stability of chromosomal DNA under salt stress [19]. Therefore, it can be seen that the non-telomeric functions of *MsTERT* are not clear and needs to be further studied. This study found that *MsTERT* not only responds to abiotic stress but also can be induced by plant hormones. After GA_3_ treatment, the expression of *MsTERT* was up-regulated with the extension of treatment time. GA_3_ is a growth hormone. *MsTERT* may also have a non-telomeric function to promote plant growth and development, which needs to be further studied.

### 3.3. Promising Indicators in Seed Germination and Seed Age Evaluation

The prediction of seed germination and seed aging is valuable for alfalfa germplasm conservation and the seed industry. In agricultural production, it is necessary to ensure that the seeds have high germination percentage, good consistency, and strong resistance [20,21]. We found that the germination percentage, which serves as a representative index of seed vigor, exhibited a positive correlation with the relative telomerase activity, telomerase reverse transcriptase (TERT) expression, and telomere length, whereas it displayed a negative correlation with aging time. However, the relative telomerase activity, telomerase reverse transcriptase (TERT) expression, telomere length, and germination percentage were negatively correlated with aging time. In the future, these indicators can potentially be applied in predicting and determining seed germination and seed aging in the alfalfa seed industry, being especially valuable in sowing and commercial transactions.

## 4. Materials and Methods

### 4.1. Preparation of Seeds

In this study, the seeds of *M. sativa* cultivar Zhongmu No.1 were harvested in the autumn of 2019 at the Gansu Forage and Pasture Research Station of the China Agricultural University (attitude 39°37′ N, longitude 98°30′ E; elevation 1480 m). Different tissue samples of root, stem, leaf, and flower were taken from flowering stage. Pod samples were taken from yellow ripening stage. The seed samples were taken from the ripe stage, and the samples removed were immediately frozen with liquid nitrogen and put into the −80 °C refrigerator for use.

### 4.2. Seed Aging Treatment and Germination Parameter Tests

Seed aging treatment was performed according to Xia et al. [22]. Briefly, seeds pre-adjusted to 10% moisture content on a fresh-weight basis were immediately sealed in an aluminum foil bag (0.12 × 0.17 m^2^, approx. 40 g in each bag) at 45 °C in a water bath. Samples were taken out every four days. The germination percentage declined to 60% on the 12th day and decreased to 0% on the 28th day. Seeds treated from day 0 to day 28 with a range of germination percentages were obtained for subsequent tests, and marked as 0, 4, 12, 20, and 28, respectively.

Germination tests were conducted according to International Seed Testing Association (ISTA, 2018) criteria, and 4 replicates were used, around 100 seeds per biological replicate. A seed was considered to be germinated if it had developed into a normal seedling. Germination percentage was the normal seedling percentage on the final day (day 10).

### 4.3. Subcellular Localization Assay

To investigate the subcellular location of *MsTERT*, vector PC-GW-Hyg-eGFP—a pre-made vector containing a GFP gene—was used to study transient gene expression. Using the primer pair 5′-ATGTCTATCTGTAGCACCGACACTTC-3′ and 5′-ATATTTGATTTTCCAAAGCAAAGAAG-3′, XbaI sites (indicated by underlining in the primer pair above) were introduced into the ORF of *MsTERT* and the resulting DNA fragment was cloned into PC-GW-Hyg-eGFP via the XbaI sites to generate the vector MsTERT–GFP. At-hook-RFP—which was used to indicate the location of nucleus—and MsTERT–GFP were injected into tobacco leaves. Tobacco leaves transfected with MsTERT–GFP were stained with Mitotracker which was used to indicate the location of mitochondria. After 24 h of expression, the leaves were observed by confocal laser scanning microscope.

### 4.4. Determination of Telomerase Activity and Telomere Length

The aged seed samples (0.2 g) from seeds imbibed for 24 h were ground into powder using liquid nitrogen, with 1.8 mL of PBS homogenate then transferred to 1.5 mL centrifuge tubes. Samples were centrifuged at 5000 rpm for 15 min and supernatants were extracted. PBS buffer solution was prepared as 0.27 g KH_2_PO_4_, 1.42 g Na_2_HPO_4_, 8 g NaCl, and 0.2 g KCl dissolved in approximately 800 mL Milli-Q water before the addition of concentrated hydrochloric acid to adjust the pH to 7.2~7.4. The obtained solution was then diluted to a volume of 1 L.

ELISA-based measurement of telomerase activity was performed using TRAPEZE ELISA Telomerase Detection Kits (TransGen Biotech, Beijing, China) [23]. Solid-phase antibody was prepared by coating microporous plates with purified plant telomerase antibody. Plant telomerase was then successively added to micropores coated with monoclonal antibody and conjugated with HRP-labeled telomerase antibody to form an antibody-antigen-labeled antibody complex. After thorough washing, TMB substrate was added for color development. The Stop Solution changes the color from blue to yellow and the intensity of the color is measured at 450 nm using a spectrophotometer, and the concentration of plant telomerase activity in the sample was calculated by the standard curve.

Telomere qPCR was performed as described by Andreja and Hudon [24,25] with the following modifications. Real-time kinetic quantitative PCR determines for each sample well the Ct, i.e., the fractional cycle number at which the well’s accumulating fluorescence crosses a set threshold that is several standard deviations above baseline fluorescence. The qPCR method measures relative telomere lengths, not absolute telomere lengths, and relative telomere length is believed to reflect the actual differences in telomere length in individuals. The amount of the gene of interest (T) is measured compared to the amount of a single copy gene (S) that is assumed to be constant. Experiments are performed using separate 96-well plates. The single copy gene used in our study was β-actin, which is an important cytoskeletal protein. Telomere length is expressed relative to the internal single gene control (β-actin) measured from the same sample of DNA. The method used was essentially the same primers developed by Aronen and Chen et al. [26,27], using the CFX96 Real-Time System (Bio-Rad, Hercules, CA, USA), a thermal cycler equipped to excite and read emissions from fluorescent molecules during each cycle of the PCR. The relative T/S ratio reflects the length differences in telomeric DNA relative to the constant β-actin amplicon and was calculated using the following formula: telomere length = 2^−∆∆Ct^ where ∆Ct = Ct_Telomere_ − Ct_β-actin_, expressed as the amount of telomere hexameric repeats, termed relative telomere length.

The crossing point of a sample depends on the initial concentration of DNA. Similar to previous studies, the DNA concentration used in this present study was 30 ng/µL and the number of cycles used was 30 for telomere repeats and 30 for β-actin gene. The concentration of β-actin was 10 µM.

### 4.5. Abiotic Stress and Hormone Treatments of Alfalfa Seeds

Alfalfa seeds of the same size with no diseases and pests were selected, disinfected with 75% ethanol for 1 min, rinsed with sterile water 5 times, treated with 2% NaCl solution for 20 min, and rinsed with sterile water 10 times [28]. The sterilized seeds were cultured in MS solid medium (20 °C, 16 h light/8 h dark). The seven-day-old seedlings were transferred to a flask containing 1/2 MS liquid medium for continuous culture (20 °C, 16 h light/8 h dark). At the 3-leaf stage, they were treated with drought (20% PEG 6000), salt (100 mmol·L^−1^ NaCl) [28], 1 mmol·L^−1^ IAA, 1 mmol·L^−1^ ABA, and 1 mmol·L^−1^ GA3. After treatment for 0, 2, 12, 24, and 72 h, respectively, the whole plant was wrapped in tin foil paper, immediately frozen with liquid nitrogen, and placed in the refrigerator at −80 °C for RNA extraction. The expression of *MsTERT* was analyzed.

### 4.6. MsTERT Bioinformatics Analysis Method

TERT gene sequences of soybean and Arabidopsis species were used as reference sequences in the alfalfa genome (https://figshare.com/articles/dataset/Medicago_sativa_genome_and_annotation_files/12623960, accessed on 11 December 2022). Blast search was carried out to screen the *MsTERT gene* sequence of alfalfa TERT. The sequence of each species was downloaded from physiotome (https://phytozome.jgi.doe.gov/pz/portal.html, accessed on 11 December 2022) and NCBI (https://www.ncbi.nlm.nih.gov, accessed on 11 December 2022). Clustal W was used to compare TERT sequences of different species, MEGA8 was used to construct phylogenetic tree (bootstrap = 1000) by Neighbor-Joining method, and *MsTERT* was analyzed by physical and chemical properties, hydrophobicity, and tertiary structure prediction by ExPASy (http://www.expasy.ch/tools/pi_tool.html, accessed on 11 December 2022). The TERT-conserved domains of different species were analyzed by online MEME website (http://meme-suite.org/tools/meme, accessed on 11 December 2022).

### 4.7. qRT-PCR and Statistical Analyses of MsTERT Gene

The RNA was extracted using Quick RNA isolation Kit (Huayueyang Biotech Co., Ltd., Beijing, China). cDNA was synthesized from 1 μg RNA using *EasyScript*^®^ All-in-One First-Strand cDNA Synthesis SuperMix for qPCR Kit (TransGen Biotech, Beijing, China). qRT-PCR analysis was performed on a CFX96 Real-Time System (Bio-Rad, Hercules, CA, USA) using 2×RealStar Green Fast Mixture (Genstar, China) with *MsTERT* gene as reference gene. According to the *MsTERT* sequence, multiple pairs of primers were designed by primer 5 for pre-experiment. After formula calculation, MsTERT-F/MsTERT-R primers (Table 2) had the highest amplification efficiency (about 95%), so this pair of primers was selected for *MsTERT* expression pattern analysis in subsequent experiments. Each sample has three technical repetitions. The thermal cycle program was 95 °C for 25 min, 40 cycles of 95 °C for 5 s, and 60 °C for 10 s. The gene expression data are calculated by 2^−∆∆Ct^ method.

### 4.8. Data Analysis and Figure Construction

Significant differences between aging treatments were calculated in SPSS 22 using ANOVA and Duncan’s test. Pearson’s correlation coefficients of 5 indicators were obtained by using R package “corrplot”. The means and standard errors were computed, and bar charts and line charts were constructed using GraphPad Prism version 8.0.

## 5. Conclusions

In summary, the *MsTERT* gene was identified and distributed in chromosome 3 of alfalfa. The gene comprises the telomerase RNA binding domain (Telomerase-RBD) and the telomerase reverse transcriptase domain (RT), and exhibits a relative molecular mass of 138.94 kD. Tissue expression profiling revealed that *MsTERT* displayed the highest expression levels in dry seeds, followed by flowers and leaves, and the lowest expression in pods. The present investigation also discovered that seed aging could stimulate *MsTERT* expression. Notably, the germination percentage, which serves as a representative index of seed vigor, exhibited a positive correlation with the relative telomerase activity, TERT expression, and telomere length, whereas it displayed a negative correlation with aging time. However, the relative telomerase activity, TERT expression, telomere length, and germination percentage were negatively correlated with aging time. These results suggest that the relative telomerase activity, TERT expression, and telomere length could serve as valuable biomarkers of seed aging. This study provides a foundation for future investigations to establish the precise regulation of the *MsTERT* gene with regard to seed vigor in alfalfa. Additionally, the stress-responsive *MsTERT* genes may be exploited for the genetic improvement of stress resistance and seed vigor in alfalfa.

## Figures and Tables

**Table 1:** Cis-regulatory elements in the promoter of *MsTERT* gene.

Regulatory Elements	Strand	Number	Sequence	Function
CAAT-box	+ −	20	CAAAT CCAAT	Common cis-acting elements in promoter and enhancer regions
TATA-box	+ −	63	ATATAA ATTATA TACAAAA TACATAAA TATA TATAA TATAAA TATAAAA TATAAAT TATACA TATATA TATATAA TATTTAAA	Core promoter elements around −30 of transcription start
LTR	+ −	3	CCGAAA	Cis-acting element involved in low-temperature responsiveness
MBS	−	1	CAACTG	MYB binding site involved in drought-inducibility
ARE	+ −	3	AAACCA	Cis-acting regulatory element essential for the anaerobic induction
TATC-box	+	1	TATCCCA	Cis-acting element involved in gibberellin-responsiveness
TGA	−	1	AACGAC	Auxin-responsive element
TCA	+	2	CCATCTTTTT	Cis-acting element involved in salicylic acid responsiveness

**Table 2:** Primers of *MsTERT* used in RT-qPCR.

Gene	Primer Name	Primer Sequence (5′-3′)
*MsTERT*	MsTERT-F	CAGGGTTGGAGATGATTA
	MsTERT-R	TAGAGACTGATTGGAGGA
Actin	Actin-F	CAAAAGATGGCAGATGCTGAGGAT
	Actin-R	CATGACACCAGTATGAGAGGTCG

## Data Availability

Data are contained within the article.
